# 2D or Not 2D? Impact of Bulky Cation Deposition Method on Inverted Perovskite Solar Cells

**DOI:** 10.1021/acsami.5c22628

**Published:** 2026-04-01

**Authors:** Marielle Deconinck, Shivam Singh, Vladimir Shilovskikh, L. Andrés Guerrero-León, Boris Rivkin, Yana Vaynzof

**Affiliations:** † Chair for Emerging Electronic Technologies, 9169 Technical University of Dresden, Nöthnitzer Str. 61, Dresden 01187, Germany; ‡ Leibniz-Institute for Solid State and Materials Research Dresden, Helmholtzstraße 20, Dresden 01069, Germany

**Keywords:** 3D perovskite, 2D perovskite, passivation, interlayer, cathodoluminescence, interfacial modification, inverted architecture solar cell, defect passivation

## Abstract

A three-dimensional/two-dimensional (3D/2D) heterojunction interface is well-known for reducing surface recombination in perovskite solar cells. The effectiveness of 3D/2D interfaces has been particularly highlighted in standard (n-i-p) structures. However, their use in inverted (p-i-n) structures is less common due to limitations on the thickness of the 2D phase, which is essential for efficient electron extraction. In this study, we investigate whether 3*D*/2D dimensional engineering is beneficial in inverted architecture devices by introducing the bulky organic cation 4-chlorophenethylammonium iodide (Cl-PEAI) to triple cation Cs_0.05_(MA_0.17_FA_0.83_)_0.95_Pb­(I_0.9_Br_0.1_)_3_ perovskite films using two different deposition methods: as a separate interlayer on top of the fully formed 3D perovskite and via an antisolvent approach during film formation. Through low-energy cathodoluminescence measurements—a highly sensitive method for probing the presence of 2D phases on the surface of 3D layers—we reveal that the interlayer method leads to the formation of 2D domains (*n* = 1 and *n* = 2). In contrast, the antisolvent method results in surface and grain boundary passivation without the formation of a detectable separate 2D phase. This observation is further correlated with device performance, where passivation leads to improvement, while 2D phase formation does not. These results highlight the significant impact of the deposition method on the formation of the 2D layer over the 3D perovskite in inverted architectures, revealing that 2D phase formation is not always beneficial for device performance in inverted architecture solar cells.

## Graphical abstract

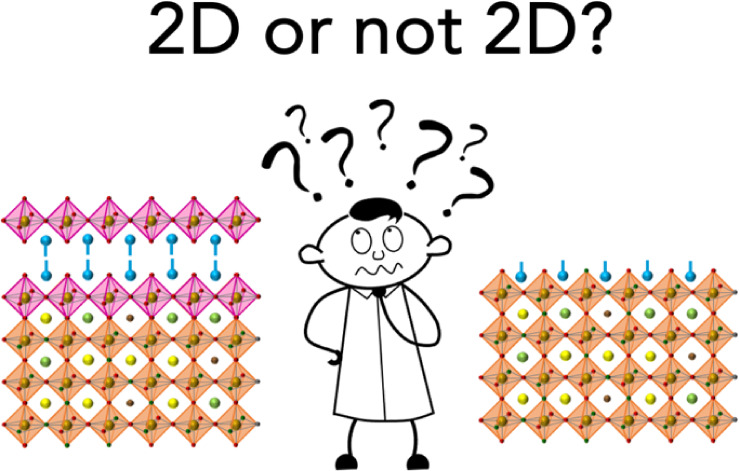

## 1 Introduction

Metal-halide perovskites (MHPs) have emerged as highly promising materials for a wide range of photovoltaic applications, including photovoltaics, light emitting diodes, thin-film transistors and memory devices, due to their exceptional optoelectronic properties, including strong light absorption across the visible spectrum, long carrier diffusion lengths, high defect tolerance, and tunable bandgaps, which can be precisely engineered through compositional and structural modifications.^1–6^ Rapid progress in materials and device engineering has enabled perovskite solar cells (PSCs) to achieve power conversion efficiencies (PCEs) exceeding 27%, putting them on par with conventional silicon solar cells and attracting widespread interest in both academia and industry.^7^

This significant rise in efficiency has been a result of substantial advances in materials chemistry, device architecture, and interface engineering.^8–11^ Among the critical factors influencing performance, defect passivation plays a central role.^12,13^ Structural imperfections, including vacancies, grain boundaries, and surface states, often act as nonradiative recombination centers.^14,15^ These defects limit carrier lifetimes and reduce the open-circuit voltage (*V*_OC_), constraining overall device efficiency.^16–19^ Consequently, understanding defect passivation has become a key research focus in the development of high-performance PSCs.^20–22^

Various strategies have been explored to mitigate the effects of defects. Small organic molecules such as phenethylammonium iodide (PEAI) and formamidinium acetate (FAAc) have been shown to enhance carrier transport and reduce the trap-state density.^23–25^ Polymeric passivators, such as poly­(methyl methacrylate) (PMMA) and polyethylenimine (PEI), can in addition improve film uniformity and serve as barriers to environmental degradation.^26,27^ Furthermore, inorganic additives such as potassium iodide (KI), rubidium bromide (RbBr), and cesium salts suppress ion vacancies, thereby improving crystallinity and charge transport propertie.^28–31^

One particularly effective strategy for enhancing efficiency and stability is the incorporation of two-dimensional (2D) perovskite phases on top of conventional three-dimensional (3D) perovskite film.^32^ Here, “2D” refers to layered Ruddlesden–Popper (RP) structures consisting of inorganic lead octahedra sheets separated by bulky organic cations and refers to the nature of electronic confinement in the system rather than the thickness of the layer. This results in the formation of 3D/2D heterostructures, which can simultaneously passivate defects, suppress ion migration, and increase moisture resistance.^33,34^ For example, bulky organic cations such as phenethylammonium (PEA^+^) and butylammonium iodide (BA^+^) can form low-dimensional RP phases, which provide surface protection and modify interfacial energetics.^35–37^ Additionally, alkali metal cations such as rubidium and cesium have been shown to enhance lattice stability and reduce phase segregation, thereby contributing to improved device longevity.^38,39^

Recent progress has further advanced this strategy by engineering more complex 3*D*/2D heterostructures, leading to significant improvements in both efficiency and operational stability. For instance, the incorporation of inorganic dopants such as Nb^5+^ into the 2D capping layer has been shown to enhance carrier transport and reduce defect density, resulting in devices with PCEs above 23%.^40^ Dual-interface designs that sandwich the 3D perovskite layer between two in situ grown 2D layers (2D/3D/2D) have demonstrated improved mechanical resilience, reduced internal stress, and enhanced long-term operational stability, with PCEs exceeding 24% on rigid substrates and over 21% on flexible ones.^41^ Solvent-engineered post-treatments, such as those using meta-amidinopyridine, have enabled the formation of highly ordered 2D phases with superior crystallinity and moisture resistance, achieving certified PCEs of ∼25.4% and maintaining over 80% performance after 1000 h under damp heat conditions.^42^ Additional studies have shown that careful tuning of the 2D layer thickness, for example by incorporating *n* = 3 Ruddlesden–Popper phases, can optimize interfacial energetics, increase open-circuit voltage and fill factor, and reduce hysteresis.^43^ Together, these developments demonstrate the growing versatility of 3*D*/2D perovskite heterostructures in addressing the key challenges of performance and long-term reliability.

While the benefits of bulky organic cation incorporation are well-documented in conventional n-i-p PSC architectures, their role in inverted p-i-n configurations remains less thoroughly investigated.^44^ In standard n-i-p devices, the introduction of bulky organic cations, such as PEAI and its halogenated derivatives, has been shown to passivate surface defects effectively and form stable 3D/2D heterojunctions, thereby improving carrier dynamics and device stability. For instance, Kodalle et al. demonstrated that incorporating 4-chlorophenethylammonium iodide (Cl-PEAI) during perovskite crystallization promotes the formation of a well-defined 3D/2D interface, leading to enhanced film morphology, reduced nonradiative recombination, and improved PCE in an n-i-p device.^45^ Inverted devices offer several advantages, including reduced hysteresis, better compatibility with flexible substrates, and simplified fabrication processes.^41^ However, the implementation of 3D/2D heterostructures in p-i-n devices presents unique challenges, primarily due to the limitations imposed by the thickness of the 2D phase. Unlike n-i-p architectures, where thicker 2D layers can be tolerated, in p-i-n configurations, excessively thick 2D capping layers tend to hinder efficient charge extraction by increasing the series resistance and creating energetic barriers for carrier transport.^46,47^ This restricts the permissible thickness of the 2D layer, making it difficult to form well-defined 2D perovskite phases without compromising device performance. As a consequence, it is not yet fully understood whether bulky organic cations in p-i-n devices form 2D perovskite phases or act primarily as surface or interfacial passivators.^48,49^ This distinction is essential for designing effective passivation strategies tailored to inverted PSC structures.

In this context, we investigate the application of Cl-PEAI, a halogenated derivative of the widely studied PEAI molecule,^49–51^ as a bulky organic cation deposited atop of 3D perovskite in inverted architectures. Cl-PEAI is an organic ammonium salt capable of reacting with excess lead iodide (PbI_2_) in perovskite films to form low-dimensional RP phases.^46^ In these structures, the large Cl-PEA^+^ cations insert between layers of lead halide octahedra, disrupting the three-dimensional lattice and promoting the formation of 2D perovskite domains. These domains can effectively passivate surface defects, reduce nonradiative recombination, and improve resistance to environmental degradation. Furthermore, the chlorine substituent may influence film morphology and interfacial energetics to enhance performance relative to nonhalogenated analogs.^52^

In this study, we systematically investigate the role of Cl-PEAI in inverted p-i-n perovskite solar cells by comparing two widely used deposition methods: (i) postdeposition spin-coating onto fully formed 3D perovskite films and (ii) incorporation during the antisolvent dripping step in the film formation process. In both approaches, the Cl-PEAI and/or the formed 2D phases are not used as electron-transport layers but rather as interfacial modifiers at the perovskite/PCBM interface. We vary the Cl-PEAI concentration for each approach to understand how processing conditions influence the extent of 2D phase formation, defect passivation, and interfacial quality. A comprehensive suite of characterization techniques, including X-ray diffraction (XRD), photoluminescence (PL), and cathodoluminescence (CL), is employed to differentiate between the formation of true 2D perovskite phases and simple surface interactions. We then correlate these findings with device metrics such as PCE, *V*_OC_, and stability under environmental stress, providing deeper insight into the function of halogenated bulky organic cations in inverted PSCs.

## 2 Experimental Details

### 2.1 Materials

Precut glass 12 × 12 mm^2^ substrates with a precoated central stripe of ITO by Psiotec Ltd. were used as a substrate for device fabrication. Glass microscope slides by Epredia were cut into 12 × 12 mm^2^ glass substrates for nonconductive characterization purposes.

The perovskite precursor solution was prepared with PbI_2_, PbBr_2_ and CsI from TCI, and MAI (CH_3_NH_3_I) and FAI (HC­(NH_2_)_2_I) from Greatcell Solar Materials. Cl-PEAI (C_8_H_10_ClNI) was also purchased from Greatcell Solar Materials. PCBM was purchased from Lumtec, and PTAA and BCP from Sigma-Aldrich. All the anhydrous solvents were purchased from Acros Organics. Silver pellets for thermal evaporation of the top contact were purchased from Kurt J. Lesker Company.

### 2.2 Solution Preparation

PTAA was used to form a hole-transport layer (HTL). 1.5 mg was dissolved in 1 mL of anhydrous toluene and kept overnight stirring at room temperature. The perovskite precursor solution (1.2M) was prepared using mixed cations (Pb, Cs, FA and MA) and halides (I and Br) dissolved in a solvent mixture of DMF/DMSO (4:1) according to the formula of Cs_0.05_(MA_0.17_FA_0.83_)_0.95_Pb­(I_0.83_Br_0.17_)_3_ with an excess of 1% PbI_2_. The PCBM solution was dissolved in anhydrous chlorobenzene with a concentration of 20 mg mL^–1^ and kept overnight, stirring at 70 °C, before being filtered through a 0.22 μm polytetrafluoroethylene (PTFE) filter before use. BCP was dissolved in isopropanol at a concentration of 0.5 mg mL^–1^ and kept overnight, stirring at 70 °C. Cl-PEAI stock solutions (20 mM) were prepared by dissolving the salt in IPA for the interlayer method and in a CB/IPA (9/1) mixture for the antisolvent method. These stock solutions were subsequently diluted with the respective solvents to obtain the working concentrations used in this study.

### 2.3 Device and Film Fabrication

The substrates were cleaned sequentially in an ultrasonic bath with deionized water, acetone, and IPA for 15 min each. Substrates were dried with a stream of nitrogen gas and treated with oxygen plasma for 10 min at 100 mW. The substrates were immediately transferred to a drybox (relative humidity <2%) for HTL, perovskite, and Cl-PEAI deposition. The 35 μL of PTAA solution was spin-coated at 2000 rpm for 30 s, with an acceleration of 1000 rpm s^–1^, onto each substrate. The spin-coated substrates were then annealed immediately at 100 °C for 10 min. After a 5 min cool-down, the substrates were each treated with a 35 μL wash of DMF solution, spin-coated at 3000 rpm for 30 s with an acceleration of 1500 rpm s^–1^.

The perovskite solution was deposited with a volume of 40 μL via a two-step spin-coating procedure with 1000 rpm for 10 s and 6000 rpm for 30 s. The antisolvent (either pure CB or, for the antisolvent method, Cl-PEAI dissolved in CB/IPA) was rapidly dripped onto the spinning substrate at a volume of 175 μL during the last 5 s of the second spin-coating step. The perovskite samples were then annealed at 100 °C for 30 min. For the interlayer method, 35 μL of the Cl-PEAI solution was dynamically spin-coated at 3000 rpm for 30 s with an acceleration of 1500 rpm s^–1^, before annealing on a hot plate at 100 °C for 10 min.

For the ETL deposition, the perovskite films were transferred to a nitrogen-filled glovebox. The PCBM solution was spin-coated statically at 2000 rpm for 30 s, with an acceleration of 1000 rpm s^–1^. The PCBM-coated films were kept bench dry for 10 min. Subsequently, the BCP solution was statically spin-coated at 4000 rpm for 30 s, with an acceleration of 1000 rpm s^–1^, as a hole-blocking layer. Finally, 80 nm Ag was thermally evaporated onto the samples under a vacuum of 4 × 10^–7^ mbar. The device area was defined as 4.5 mm^2^ by a metal shadow mask.

### 2.4 Device and Film Characterization

For each characterization measurement, separate samples from the same batch were used, unless stated otherwise. The samples were stored in a nitrogen-filled glovebox and in the dark until each measurement.

### 2.5 X-ray Photoemission Spectroscopy

The samples were transported to a separate lab in the same building in air and covered with aluminum foil as rapidly as possible for XPS measurements. The samples were transferred to an ultrahigh-vacuum chamber (ESCALAB Xi+ by Thermo Scientific, base pressure: 2 × 10^–10^ mbar). XPS measurements were carried out using an XR6 monochromated Al Kα source (hν = 1486.6 eV) and a pass energy of 20 eV.

### 2.6 Scanning Electron Microscopy

The samples were transported to a separate lab in an adjacent building in air and covered with aluminum foil as rapidly as possible for SEM measurements. High-resolution images of perovskite films were acquired with a Zeiss Gemini 500 scanning electron microscope (SEM), with a landing energy of 1.5 kV for imaging or 0.5 kV for surface-sensitive imaging.

### 2.7 Photoluminescence Spectroscopy

The samples were transported to a separate lab in the same building in air and covered with aluminum foil as rapidly as possible for PL measurements. PL spectra were recorded using an Edinburgh Instruments FSP 920 spectrometer equipped with a Xenon (Xe) lamp as the excitation source. The excitation monochromator slit width was set to 6 nm, while the emission monochromator slit width was set to 10 nm. Measurements were performed using an excitation wavelength of 460 nm.

### 2.8 Cathodoluminescence Spectroscopy

The samples were transported to a separate lab in a building close-by in air and covered with aluminum foil as rapidly as possible for CL measurements. CL measurements were performed using a FE-SEM Zeiss Ultra55 equipped with a CL setup, consisting of a parabolic mirror, a Czerny-Turner monochromator, and an on-axis mounted Peltier cooled CCD camera S7031–1006 from Hamamatsu (back illuminated, 1,024 × 64 pixels). Spectra were recorded for the wavelengths between 450 and 850 nm with a blaze grating of 300 lines/mm and a blaze wavelength of 500 nm at electron accelerating voltages of 0.3–10 kV for probing the luminescence of the material at different depths. Instrumental broadening in the experimental setup was 17 nm.

### 2.9 X-ray Diffraction

The samples were transported to a separate lab in an adjacent building in air and covered with aluminum foil as rapidly as possible for XRD measurements. The X-ray diffraction measurements were performed in ambient air. The measurement system was a Bruker D8 Discover with a Lynxeye 1D detector.

### 2.10 UV–vis Absorption

The samples were transported to a separate lab in an adjacent building in air and covered with aluminum foil as rapidly as possible for UV–vis measurements. A Jasco V-770 Spectrophotometer was used to determine the spectral absorption of the films. A glass substrate was used as a reference.

### 2.11 Photovoltaic Characterization

The samples were transported to a separate lab in the same building in air and covered with aluminum foil as rapidly as possible for PV measurements. Current density–voltage (J-V) measurements were performed under simulated solar illumination of AM 1.5 light, using an Abet Technologies Sun 3000 AAA solar simulator with an intensity of 100 mW cm^–2^ under ambient conditions. J-V curves were measured with a Keithley 2450 source meter, scanning from −0.05 to 1.2 V and back with a step size of 0.05 V and a dwell time of 0.1 s. The light intensity was calibrated using a Si reference cell (NIST-traceable, VLSI) and corrected by determining the spectral mismatch between the solar spectrum, the reference cell, and the device’s spectral response. Substrates contained eight pixels with an active device area of 1.5 × 3 mm^2^. Samples from a different batch were used for maximum power point (MPP) tracking measurements.

### 2.12 External Quantum Efficiency

The samples were transported to a separate lab in the same building in air and covered with aluminum foil as rapidly as possible for External Quantum Efficiency (EQE) measurements. EQE spectra were recorded using the QuantX-300 system (Newport), which employs a xenon arc lamp and operates across the 325–900 nm wavelength range. The samples used for EQE measurements were also used for PV measurements first.

## 3 Results and Discussion

### 3.1 Morphological and Structural

Top-view InLens (in-lens detector) and energy-selective backscattered electron (ESB) detector scanning electron microscopy (SEM) images of the 3D control Cs_0.05_(MA_0.17_FA_0.83_)_0.95_Pb­(I_0.83_Br_0.17_)_3_ (TrCa) perovskite films (Figure 1A–B) display a characteristic microstructure typical of TrCa perovskites. The surface consists of compact grains several hundred nanometers in size, with clearly defined grain boundaries and a small number of brighter regions attributed to residual PbI_2_. Upon incorporating the additive using the interlayer method (Figure 2C–F), distinct dark regions emerge across the surface, increasing in size and coverage with increasing concentration. As the InLens detector is sensitive to secondary electrons, the reduced signal in these areas likely reflects local variations in surface composition or conductivity associated with additive accumulation. ESB images (insets) further support this hypothesis, showing initial localization at grain boundaries and subsequent formation of larger, island-like surface features. The estimated surface coverage of these dark regions increases from less than 1% at 1 mM to approximately 2.6% at 2 mM, 31.3% at 5 mM, and reaches around 58.7% at 10 mM. As we show below, the appearance of these dark regions coincides with signatures of 2D phase formation, as evidenced by XRD, PL, and CL measurements, suggesting that these regions correspond to 2D phase islands forming on the 3D perovskite layer.

**Figure 1:**
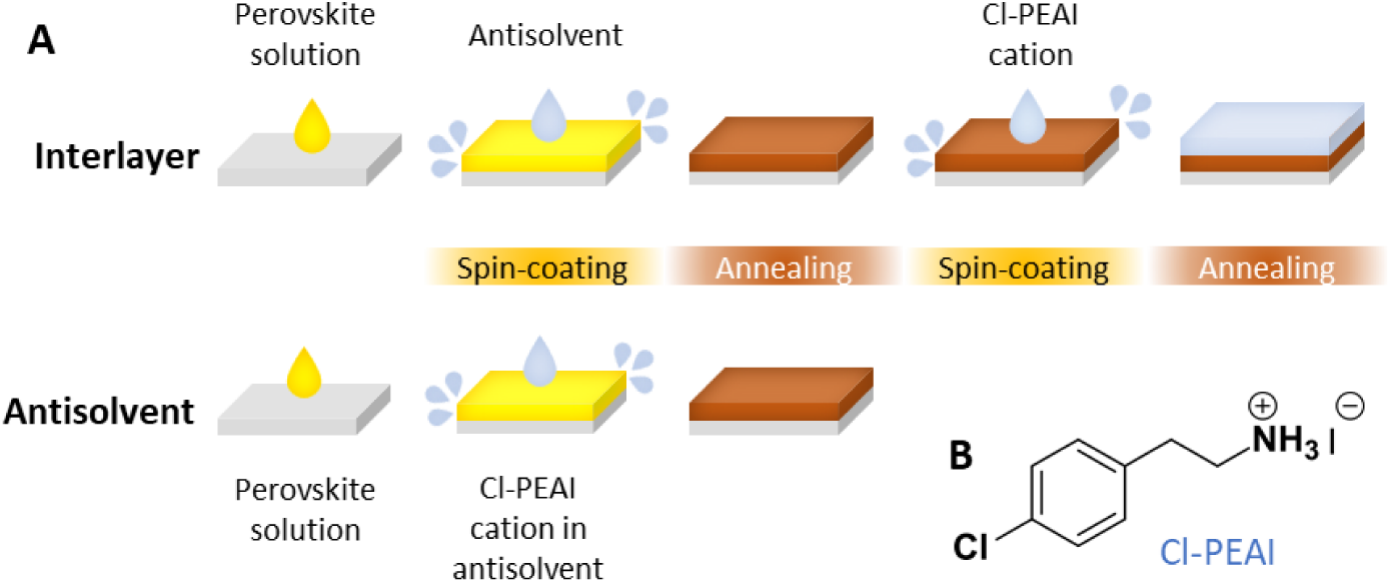
(A) Schematics of the perovskite fabrication procedure and two distinct deposition methods of the cation Cl-PEAI. (B) Chemical structure of Cl-PEAI.

**Figure 2:**
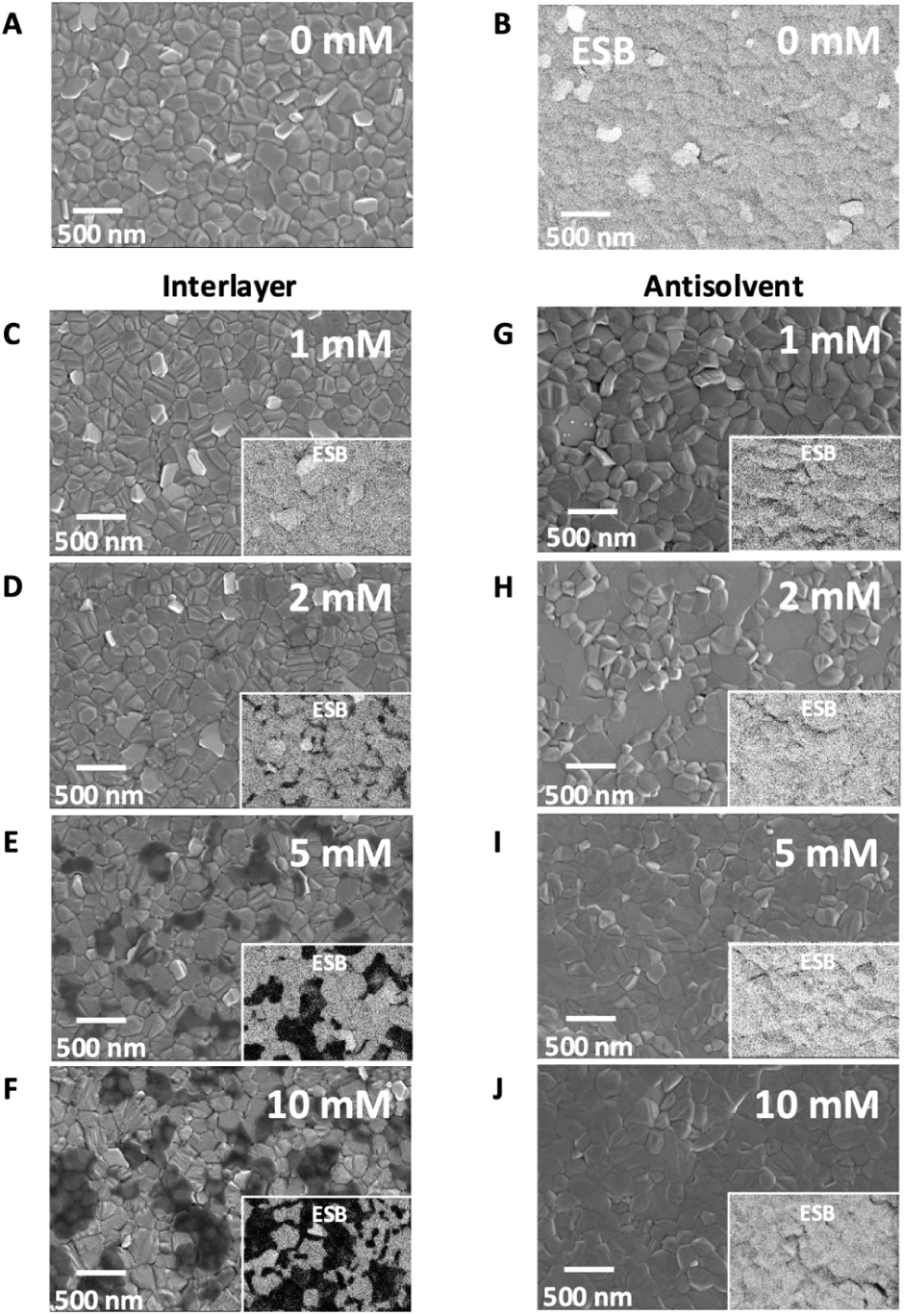
(A) InLens and (B) ESB SEM images of the reference TrCa perovskite film. (C–F) InLens SEM images of interlayer-treated TrCa films (G–J) InLens SEM images of antisolvent-treated films. The insets represent the corresponding ESB images.

In stark contrast, films in which Cl-PEAI is introduced via the antisolvent do not exhibit the dark regions seen in the interlayer approach, indicating a more uniform secondary electron yield and suggesting minimal surface compositional variation (Figure 2G–J). However, the 3D perovskite surface morphology appears to be significantly altered. The grains appear increasingly flatter, and grain boundaries become less distinct with increasing concentrations. ESB detector images (insets) reveal minimal compositional contrast, supporting the observed changes in surface texture. These results suggest that Cl-PEAI introduced via the antisolvent integrates into the surface region and grain boundaries of the 3D perovskite, modifying its morphology. In contrast, the interlayer method forms distinct Cl-PEAI-rich islands without significantly altering the underlying 3D perovskite structure.

Normalized XRD analysis (Figure 3A–B) reveals key differences in structural evolution between the two Cl-PEAI incorporation methods (raw data shown in Figure S1). In the interlayer-treated films, distinct diffraction peaks corresponding to 2D perovskite phases emerge at concentrations of 5 mM and above, consistent with the formation of low-dimensional structures.^32^ At 1 and 2 mM, no such features are observed, though the possibility of small quantities of 2D phase below the detection limit cannot be ruled out. These results suggest that a threshold concentration is required for detectable 2D phase formation via the interlayer method.

**Figure 3:**
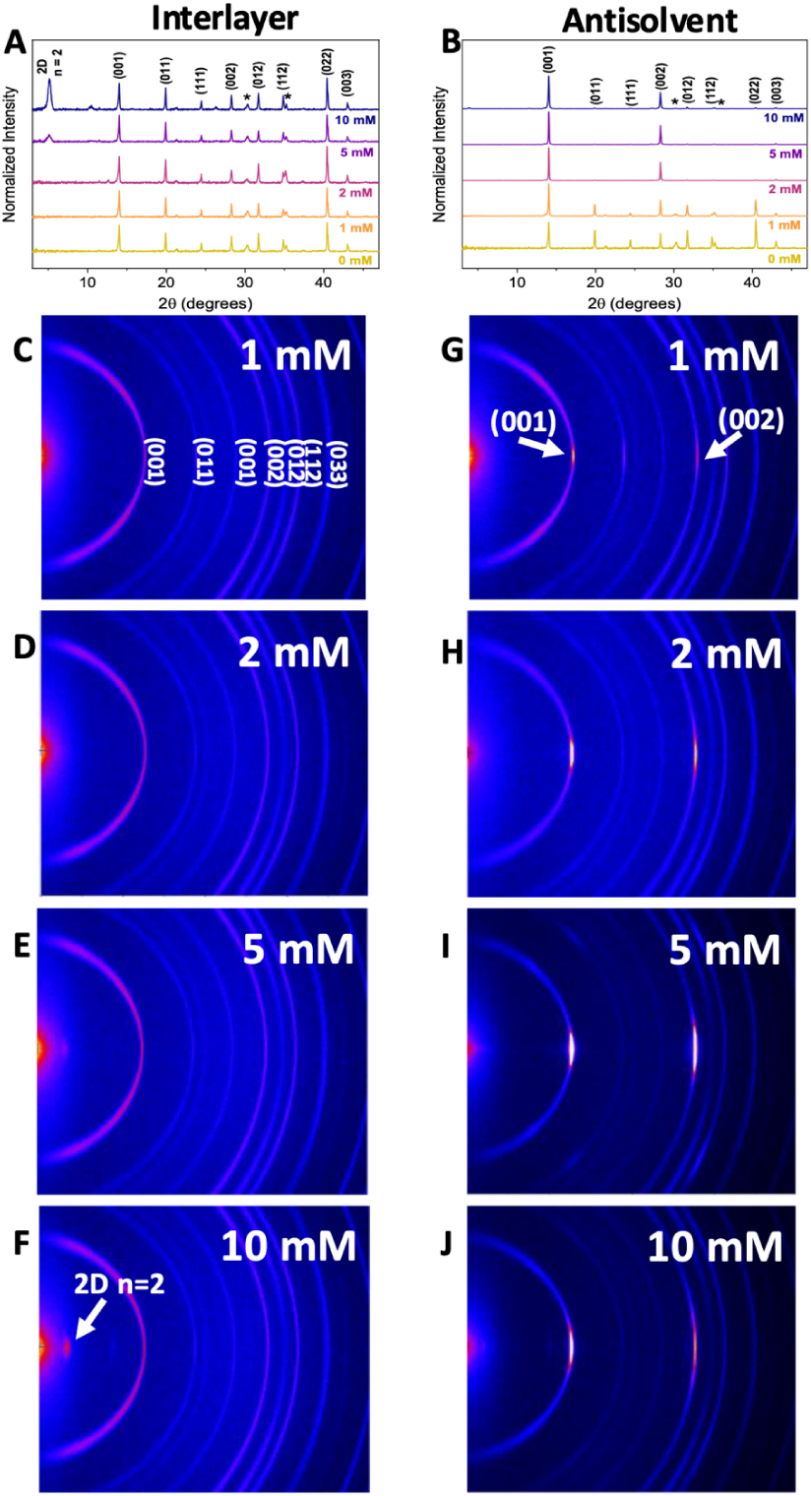
Normalized XRD patterns of (A) interlayer-treated and (B) antisolvent-treated films. 2D XRD patterns of perovskite films treated with Cl-PEAI via (C–F) the interlayer method and (G–J) the antisolvent method.

In contrast, films prepared by the antisolvent method do not show any discernible 2D-related diffraction peaks, even at higher concentrations, suggesting either the absence of 2D phases or their presence in amounts too small or disordered to be detected by conventional XRD. However, notable changes in peak intensities suggest a systematic evolution in crystal orientation. The (001) and (002) reflections become increasingly dominant with rising Cl-PEAI concentration, particularly at 5 mM, suggesting enhanced out-of-plane grain alignment. This trend diminishes slightly at 10 mM, indicating a possible saturation or disruption of alignment at higher additive loadings.

To further investigate these observations, 2D XRD patterns were acquired, as shown in Figure 3C–J. Films processed via the interlayer method (Figure 3C–F) show distinct 2D diffraction features at ≥5 mM, specifically the (001) reflection at approximately 2θ = 5.1°, confirming the formation of layered perovskite phases that are oriented parallel to the substrate. In contrast, 2D XRD of the antisolvent-treated films (Figure 3G–J) detects no such features, but instead shows stronger intensity along the (001) and (002) directions, consistent with improved vertical orientation. The strongest preferential alignment is observed at 5 mM, with a slight reduction at 10 mM, aligning with trends observed in the 1D patterns.

The improvement in orientation observed in the antisolvent method might initially be attributed to the presence of isopropanol (IPA) in the antisolvent mixture, as previous work by Telschow et al. demonstrated that IPA can promote preferential orientation in TrCa perovskites by stabilizing oriented intermediate phases.^53^ However, since our antisolvent mixture contains only 10% IPA diluted in chlorobenzene (9:1) with Cl-PEAI, the effect of IPA alone is expected to be insignificant. Telschow et al. demonstrated that preferred orientation is observed only when the IPA content exceeds 50% of the antisolvent mixture. Our control experiments support this: films processed using the same chlorobenzene/IPA (9:1) antisolvent without Cl-PEAI, as well as samples rinsed with IPA using the interlayer method (albeit without Cl-PEAI), showed no significant differences in orientation compared to untreated control films (Figure S2). These results confirm that the enhanced vertical alignment in the antisolvent-treated films arises specifically from the incorporation of Cl-PEAI and not from IPA alone.

### 3.2 Optoelectronic Signatures and Phase Identification

We conducted a series of optical measurements to gain a deeper understanding of the nature of Cl-PEAI-induced modifications, particularly the potential formation of 2D perovskite phases. A clear signature of 2D phases is observed via PL spectroscopy measured in reflection geometry when Cl-PEAI is deposited as a separate interlayer (Figure 4A). Specifically, two distinct peaks appear in the absorption spectra, corresponding to the *n* = 1 and *n* = 2 2D perovskite phases, located at approximately 520 and 570 nm, respectively. The 2D signal first becomes detectable at a Cl-PEAI concentration of 2 mM, where a weak *n* = 2 peak is visible, while no *n* = 1 peak is present. The intensity of the *n* = 2 peak reaches a maximum at 5 mM and then decreases with further increases in concentration. In contrast, the *n* = 1 peak becomes visible at 5 mM and continues increasing with higher Cl-PEAI concentrations. These results suggest a sequential formation of 2D phases, with the *n* = 2 phase forming initially at lower Cl-PEAI concentrations, followed by the emergence and growth of the *n* = 1 phase at higher concentrations. This concentration-dependent phase evolution may be understood in terms of the relative thermodynamics and kinetics of 2D phase formation. While the *n* = 1 phase is known to be thermodynamically the most stable Ruddlesden–Popper configuration, its higher nucleation barrier can delay its initial formation.^56^ In contrast, the *n* = 2 phase exhibits lower nucleation energy, allowing it to form preferentially at lower Cl-PEAI concentrations, potentially as a continuous quasi-2D (Q2D) layer atop the 3D perovskite surface. As the Cl-PEAI concentration increases beyond ∼5 mM, the conditions favor nucleation and subsequent growth of the *n* = 1 phase, which may increasingly consume the available Cl-PEAI and outcompete the *n* = 2 phase due to its greater thermodynamic driving force.^57^

**Figure 4:**
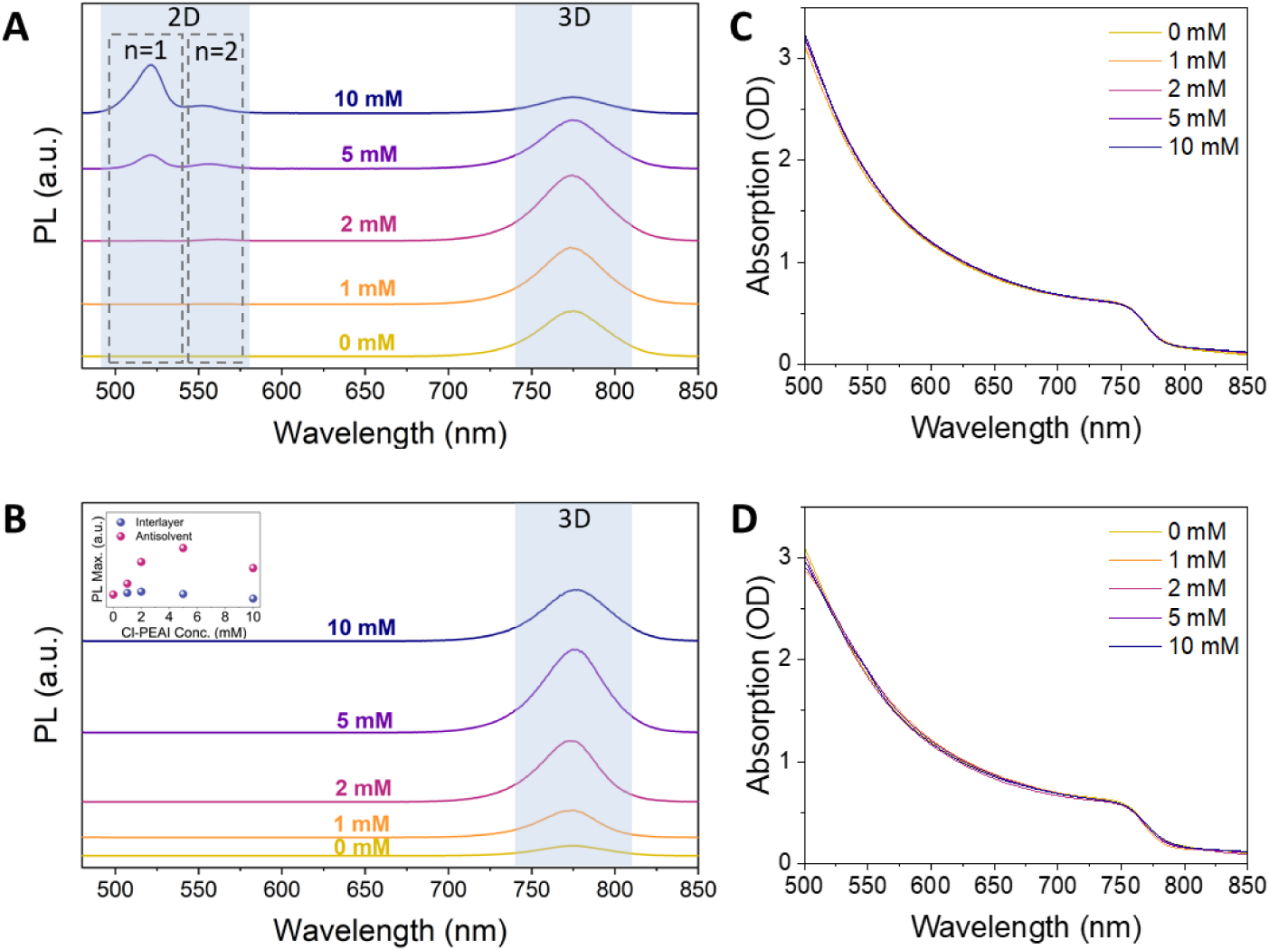
PL spectra of (A) interlayer-treated films and (B) antisolvent-treated films at different Cl-PEAI concentrations. The inset represents the 3D emission peak intensity as a function of Cl-PEAI concentration for both methods. (C) UV–vis spectra of (C) interlayer-treated films and (D) antisolvent-treated films.

Conversely, when measuring PL on these same samples using an integrating sphere instead of a reflection geometry, the 2D-related signal is not detected (see Figure S3). This absence arises from a combination of effects: multiple reflections within the sphere and reabsorption of photons in the 3D phase, which effectively mask the 2D emission, and the extreme surface confinement of the 2D layer, which limits its contribution when the collected signal averages over the entire film volume. The strong 2D peak observed in reflection PL confirms that the layer itself is highly emissive at the surface, further supporting that the 2D phase is ultrathin and localized at the topmost region of the film.

In contrast, PL spectra of films prepared by the antisolvent method show no detectable 2D phase signatures, even at high Cl-PEAI concentrations (Figure 4B). To complement these findings, UV–vis absorption spectroscopy was performed on all samples (Figure 4C–D). No features associated with 2D perovskite phases were detected in any of the UV–vis spectra, regardless of treatment method or Cl-PEAI concentration. This suggests that the 2D phases formed in the interlayer method are likely confined to the near-surface region and present in amounts below the UV–vis detection limit, highlighting the greater sensitivity of PL for detecting such subtle surface features.

Taken together with SEM observations, these results support the interpretation that the interlayer method promotes the formation of a distinct 2D perovskite overlayer, likely arising from the reaction of Cl-PEAI with residual PbI_2_ at the surface. In contrast, the antisolvent method results in surface passivation of the 3D layer using Cl-PEAI, without generating a detectable separate 2D layer.

To evaluate the impact of the two Cl-PEAI incorporation methods on optoelectronic performance, we analyzed the PL intensity of the 3D perovskite emission peaks across different concentrations (Figure 4B, inset). Overall, the PL output is significantly higher for films processed using the antisolvent method. The maximum PL intensity, defined here as the highest measured peak height of the 3D emission, is observed at a Cl-PEAI concentration of 5 mM, although all tested concentrations yield higher PL output than the control film. In contrast, the interlayer approach yields only a modest enhancement in PL, with the highest intensity observed at 2 mM, which is only slightly higher than that of the reference. At the highest concentration studied (10 mM), the interlayer approach results in PL quenching, as evidenced by a reduction in the 3D emission peak below that of the reference film, likely due to increased nonradiative recombination at high CL-PEAI coverage. These findings suggest that the antisolvent method improves the optoelectronic quality of the 3D perovskite film. In contrast, the interlayer approach offers limited benefit and can be detrimental at higher Cl-PEAI concentrations.

To gain a deeper understanding of the formation and spatial distribution of 2D perovskite phases induced by Cl-PEAI incorporation, it is crucial to employ characterization techniques with tunable and high surface sensitivity. While PL spectroscopy is widely used to identify phase signatures, its relatively large excitation volume limits its ability to detect ultrathin 2D layers confined near the surface. Cathodoluminescence (CL) spectroscopy, which utilizes an electron beam with a tunable accelerating voltage, offers enhanced surface sensitivity and spatial resolution, making it an ideal complementary technique for probing these thin 2D phases within perovskite films.

To quantify the surface sensitivity of CL spectroscopy at different electron beam energies, electron beam interaction simulations were performed to model the excitation depth within the perovskite films (Figure 5A). These results show that at accelerating voltages between 0.4 and 1 kV, the majority of the CL signal originates from within the top 25 nm of the film, while higher energies probe deeper into the bulk. Figure 5B presents a complementary visualization of the excitation profiles, illustrating the electron interaction volume as a function of depth at various beam energies, further highlighting the enhanced surface sensitivity at low voltages.

**Figure 5:**
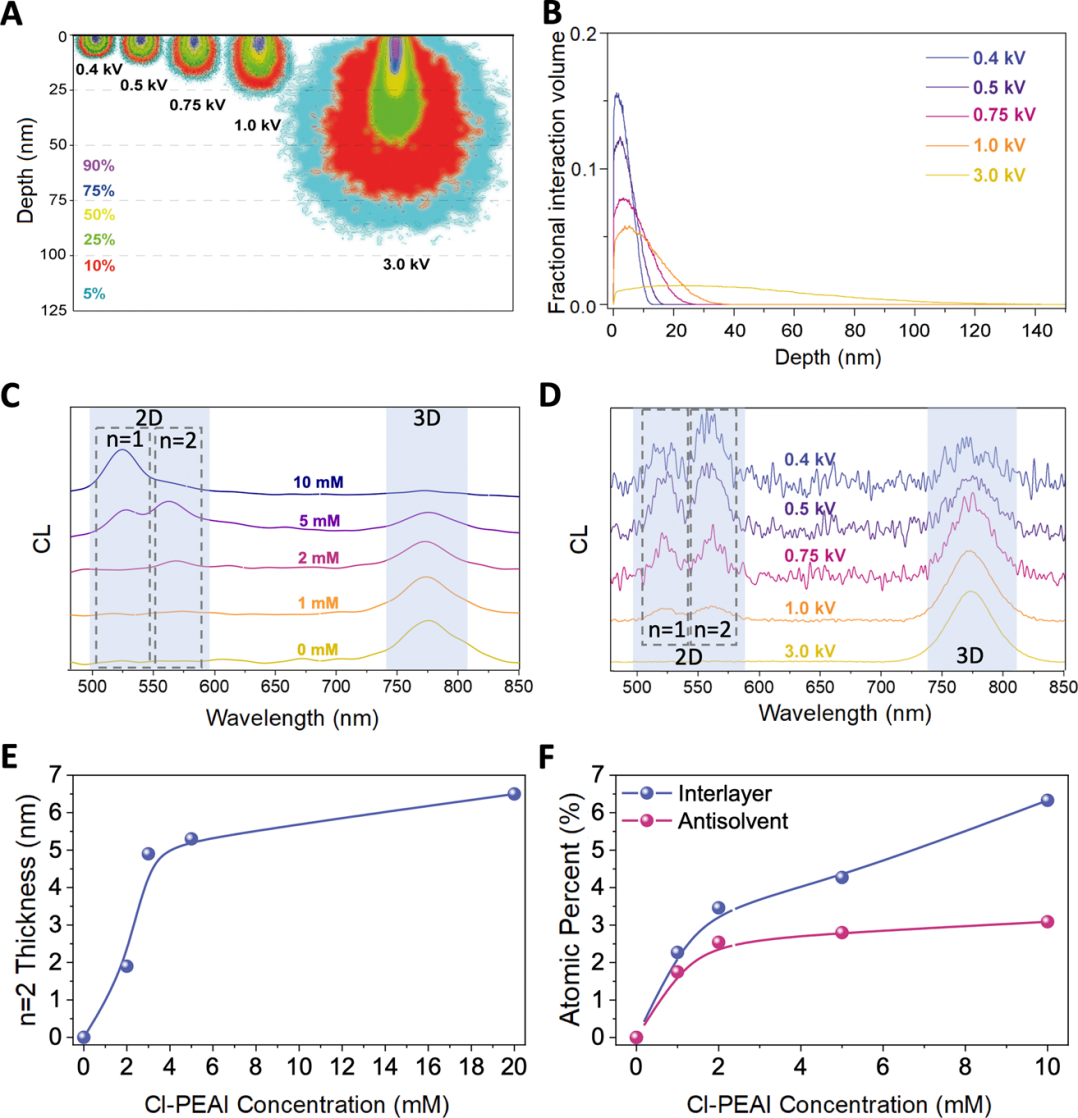
Cathodoluminescence (CL) analysis of 2D perovskite formation for the interlayer method. (A) Electron beam interaction volume simulations showing excitation depth at various voltages. (B) Simulated excitation profiles as a function of excitation depth. (C) CL spectra at 0.5 kV for different Cl-PEAI concentrations. (D) CL spectra of the 2 mM sample measured at different beam energies. (E) Estimated 2D layer thickness as a function of Cl-PEAI concentration. (F) XPS surface atomic percentage of chlorine for both interlayer and antisolvent methods.

Building on these findings, CL spectroscopy was used as a complementary and more surface-sensitive probe compared to PL to detect 2D phases in films prepared via the interlayer approach. A clear 2D signature is observed (Figure 5A), with CL able to detect smaller quantities of the *n* = 2 phase than PL alone. Notably, a weak *n* = 2 peak appears at a Cl-PEAI concentration of 1 mM, below the PL detection threshold. As the concentration increases, both *n* = 1 and *n* = 2 peaks intensify, reaching their strongest signals around 5 mM. These spectra were recorded at a beam energy of 0.5 kV, underscoring the shallow excitation depth necessary to observe these surface-confined phases.

To further explore the spatial origin of the 2D signal, CL spectra were recorded at varying electron beam energies on the 2 mM interlayer-treated sample (Figure 5B). This demonstrates a key innovation: a novel low-energy CL approach capable of detecting ultrathin 2D perovskite phases invisible under standard measurement conditions. At higher energies, such as 3 kV, only the 3D perovskite emission is detected, but at accelerating voltages of 1 kV and below, distinct peaks from the *n* = 1 and *n* = 2 2D phases emerge. The relative intensity of the *n* = 2 peak increases as the beam energy decreases, confirming that these phases are localized near the film surface. Because most CL studies use higher beam energies, such ultrathin 2D features have likely gone undetected. To our knowledge, this is the first report demonstrating that tuning beam energy in CL selectively reveals ultrathin 2D perovskite phases confined to the top few nanometers, providing a powerful technique for probing surface composition and vertical phase distribution in hybrid perovskite films.

Using this information, the thickness of the 2D layer was estimated as a function of Cl-PEAI concentration (Figure 5E). The modeled thickness increases with concentration, e.g., to approximately 5.2 nm, at a concentration of 5 mM, but shows a gradual saturation trend thereafter, indicating a limit to 2D layer growth. This trend aligns well with X-ray photoelectron spectroscopy (XPS) data (Figure 5F), which shows a steady increase in surface chlorine atomic percent for the interlayer method. Corresponding Cl 2p spectra are presented in Figure S4. These findings confirm that the interlayer method leads to the formation of thin, surface-localized 2D perovskite islands, whose thickness can be tuned by the Cl-PEAI concentration.

In contrast, films treated with the antisolvent method exhibit a significantly lower chlorine surface content, which plateaus beyond 2 mM. To explore whether Cl-PEAI penetrates the film when introduced via the antisolvent method, we used XPS to examine the buried interface (i.e., the perovskite-PTAA interface) for Cl. We observe a substantial Cl signal (Figure S5), comparable to that at the surface of the layer, suggesting that Cl-PEAI can diffuse through the wet precursor form and reach the buried interface with the HTL. The results suggest that the antisolvent method primarily leads to surface passivation by Cl-PEAI, without detectable formation of 2D phases. We hypothesize that this can be related to the fact that when introduced in the antisolvent, the Cl-PEAI is unable to react with PbI_2_ to form a detectable 2D perovskite, since it is deposited on a wet perovskite precursor film during the spin-coating process. The presence of host solvents such as DMF and DMSO likely results in the formation of iodoplumbate complexes with these solvents, thereby rendering PbI_2_ unavailable for 2D phase formation. This is consistent with previous in situ X-ray characterization experiments that showed that PbI_2_ forms only during the annealing process of the spin-coated perovskite film.^52^

### 3.3 Photovoltaic Performance

To assess how these morphological and optical differences translate to device performance, solar cells were fabricated using an inverted p–i–n architecture: glass/ITO/PTAA/3D perovskite/Cl-PEAI/PCBM/BCP/Ag. Photovoltaic (PV) metrics for each method and concentration are shown in Figure 6. For each condition, two substrates were measured, each with eight solar cells. The data is displayed with the box showing the interquartile range, the line representing the median, and the whiskers denoting the maxima and minima, excluding outliers. Incorporating Cl-PEAI via the interlayer approach led to decreased PCE at all tested concentrations (1, 2, 5, and 10 mM). *J*_SC_ showed a marked decline across all concentrations (validated by the EQE measurements in Figure S6), *V*_OC_ slightly decreased but recovered near control values at 10 mM, and FF remained relatively stable. Overall, the interlayer approach resulted in reduced device performance compared to the reference.

**Figure 6:**
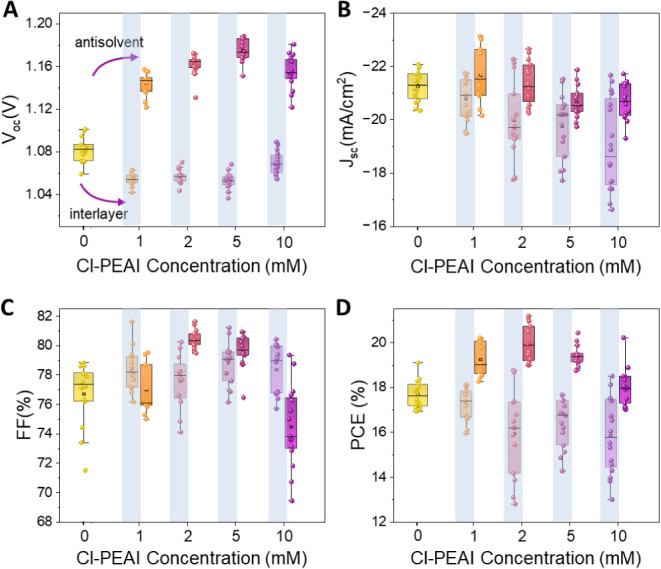
PV parameters: (A) *V*_OC_, (B) *J*_SC_, (C) FF, and (D) PCE of interlayer-treated and antisolvent-treated devices. The statistics, including the interquartile range, median, and minima and maxima excluding outliers, were calculated using two substrates per condition, with 8 solar cells per substrate.

A further increase in the Cl-PEAI concentration (up to 25 mM) introduced via the interlayer method showed that while the *V*_OC_ of the devices can be enhanced, the *J*_SC_ is reduced drastically, leading to a severe decline in the PCE (Figure S7). A further increase to 90 mM showed that concentrations above about 30 mM lead to poorly functioning devices, particularly due to a very low *J*_SC_ (Figure S8). This decline in current is likely due to the formation of a compact 2D layer on top of the 3D perovskite, which prevents efficient electron extraction. This is confirmed by top-view and cross-section SEM images of the 20 mM sample, in which, at this concentration, a continuous 2D layer forms (Figure S9).

In contrast, the addition of Cl-PEAI via the antisolvent method yielded improved results. The average PCE increased with Cl-PEAI concentration, attaining a maximum at 2 mM, then decreased for all higher concentrations tested (5 mM and 10 mM), with the highest concentration of 10 mM yielding an average PCE that still surpassed the control. The most notable improvement was observed in *V*_OC_, which increased from 1.08 V (control) to an average of 1.18 V at 5 mM. This trend aligns with PL measurements, where maximum PL intensity significantly increased at 5 mM Cl-PEAI, indicating improved optoelectronic quality. *J*_SC_ remained relatively stable, slightly rising at 1 mM, followed by modest decreases at higher concentrations. FF did not exhibit a clear trend, with increases at 2 and 5 mM and decreases at 1 and 10 mM relative to controls. These results demonstrate that optimizing Cl-PEAI concentration via the antisolvent method can significantly enhance device performance.

Stabilized PCE measurements (Figure S10) at the maximum power point (MPP) for 120 min with 2 mM Cl-PEAI devices show that both types of modifications lead to only a slight improvement in stability as compared to the reference devices. However, in our experiments, we often observed that the antisolvent samples were more stable than the interlayer ones.

It is important to note that literature reports, such as Zanetta et al.,^58^ demonstrate that incorporating 2D perovskite phases can enhance device efficiency under different processing conditions. In particular, improved vertical orientation of 2D phases has been shown to benefit charge transport and device performance in p-i-n architectures. Our results reflect the specific conditions used here and highlight how the method of Cl-PEAI incorporation influences both morphology and device performance.

## 4 Conclusion

In summary, this study offers insights into 3*D*/2D phase engineering by integrating Cl-PEAI into the 3D perovskite (TrCa) as a separate interfacial layer and using antisolvent-assisted deposition during film formation. Structural, morphological, optical, and electronic analyses reveal that these two methods induce fundamentally different interactions with the perovskite surface, significantly impacting film structure and device performance. When applied as an interfacial layer, Cl-PEAI reacts with residual surface PbI_2_ to form a thin 2D perovskite layer, as confirmed by PL, CL, and XPS. However, this method provides limited improvements to optoelectronic properties, does not improve crystallographic orientation, and reduces device efficiency. Conversely, the addition of Cl-PEAI via an antisolvent during film growth leads to the passivation of the surface, promoting better vertical grain alignment and thereby improving the optoelectronic quality without forming a detectable separate 2D phase. The study also recommends using CL to identify 2D phases that are hard to detect with conventional optical and structural methods. Overall, our results confirm that small variations in processing strategies can lead to significant differences in structure and functionality, underscoring the critical role of precise engineering in combining multifunctional organic cations for advanced perovskite optoelectronic devices.

## Supplementary Material



## Notes

Additional figures (XRD data, 2D XRD, PL, XPS, SEM, EQE, MPPT) and experimental details (PDF)
