# Supplementary material for: 2D or Not 2D? Impact of Bulky Cation Deposition Method on Inverted Perovskite Solar Cells
Source: ACS Appl Mater Interfaces. 2026 Apr 1;18(14):20366–76. doi: 10.1021/acsami.5c22628 (PMC13088042; doi:10.1021/acsami.5c22628)
Supplement: Supplementary file 1 [file am5c22628_si_001.pdf]

Supporting Information

2D or Not 2D? Impact of Bulky Cation Deposition Method on Inverted Perovskite Solar Cells

Marielle Deconinck[1,2], Shivam Singh[1,2], Vladimir Shilovskikh[1,2], L. Andrés Guerrero-León[1,2], Boris Rivkin[1,2], Yana Vaynzof[1,2]*

1 Chair for Emerging Electronic Technologies, Technical University of Dresden, Nöthnitzer Str. 61, 01187 Dresden, Germany.

2 Leibniz-Institute for Solid State and Materials Research Dresden, Helmholtzstraße 20, 01069, Dresden, Germany.

Corresponding author: Yana Vaynzof. Email: y.vaynzof@ifw-dresden.de

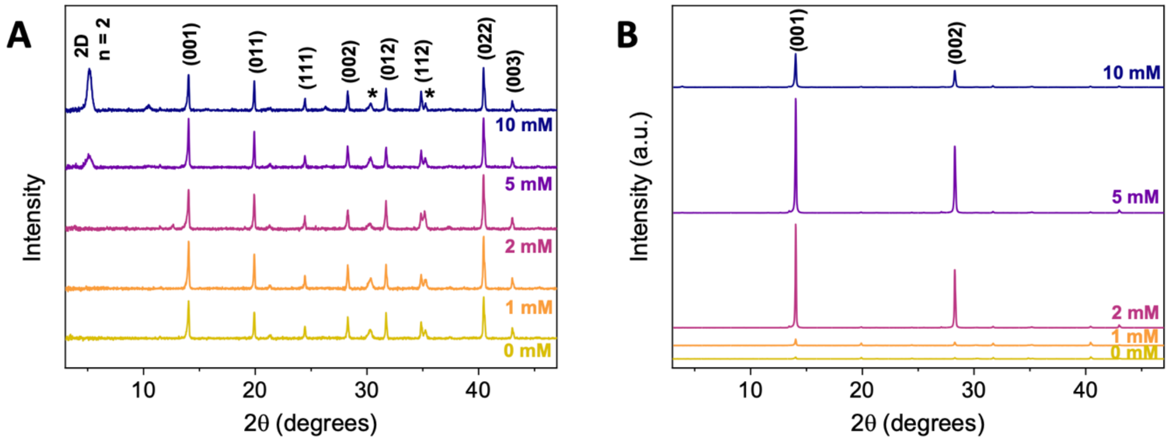

Figure S1: XRD patterns without normalization of (A) interlayer-treated and (B) antisolvent-treated films.

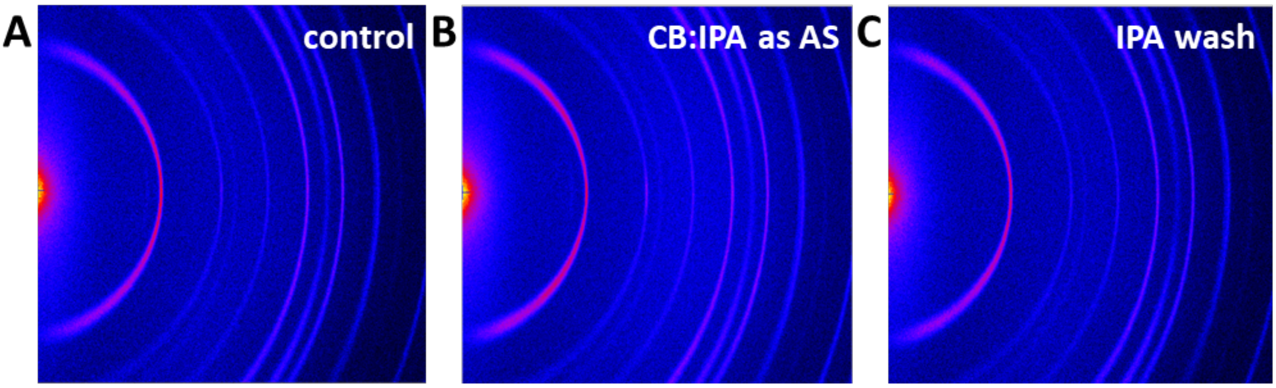

Figure S2: 2D XRD spectra of (A) the control film, (B) the control film using CB:IPA as antisolvent and (C) the control with a separate IPA wash after perovskite film formation.

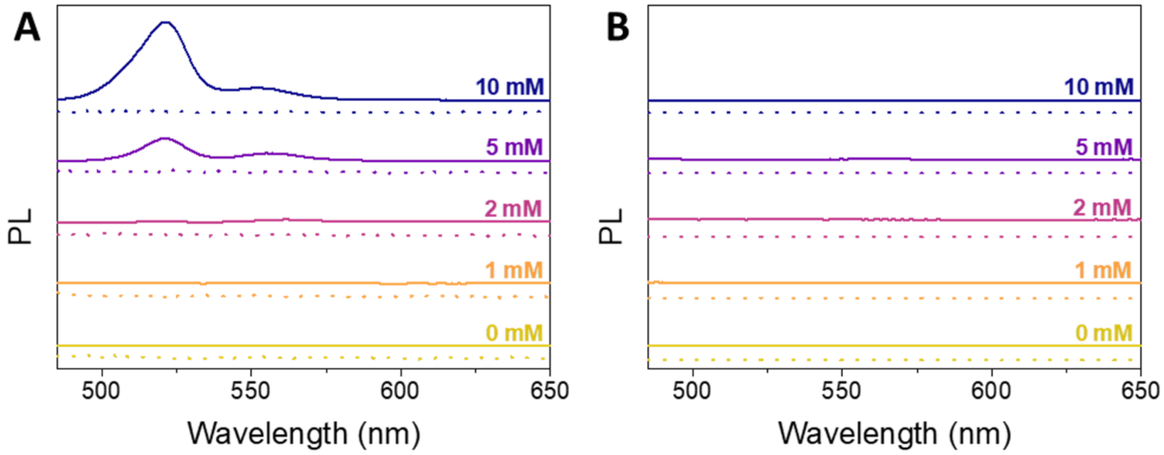

Figure S3: Comparison between PL measurements performed in a reflection geometry (continuous lines) and by means of an integrating sphere (dotted lines) using (A) the interlayer and (B) the antisolvent method.

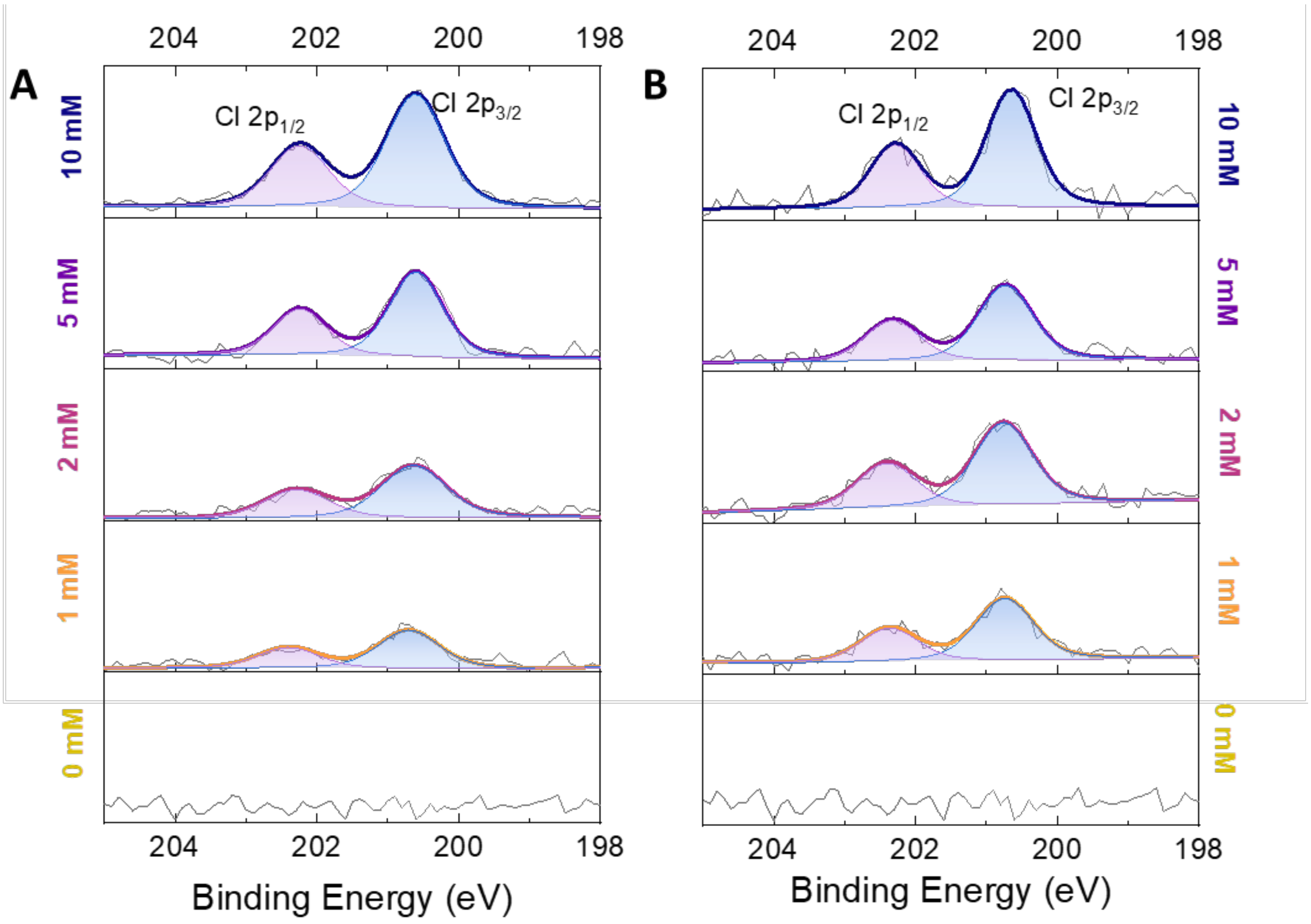

Figure S4: Cl 2p spectra for films using (A) the interlayer and (B) the antisolvent method.

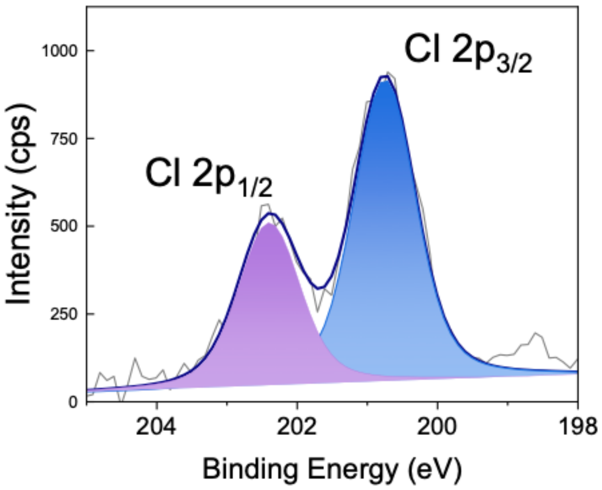

Figure S5: Cl 2p spectrum measured at the buried interface of a perovskite layer prepared using the antisolvent method with 10 mM concentration of Cl-PEAI.

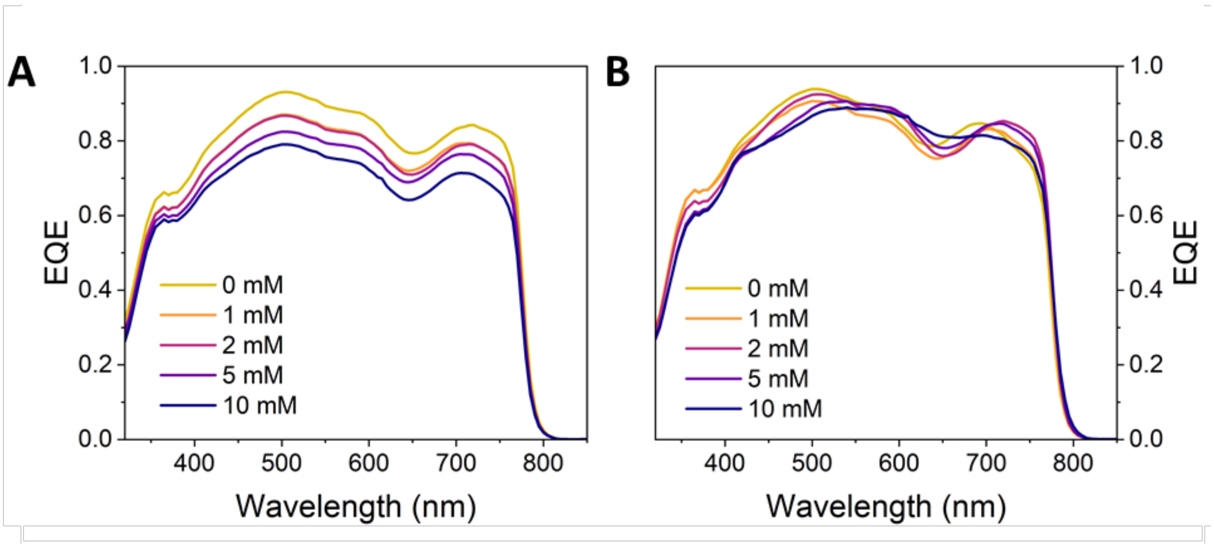

Figure S6: EQE spectra of devices using (A) the interlayer and (B) the antisolvent method.

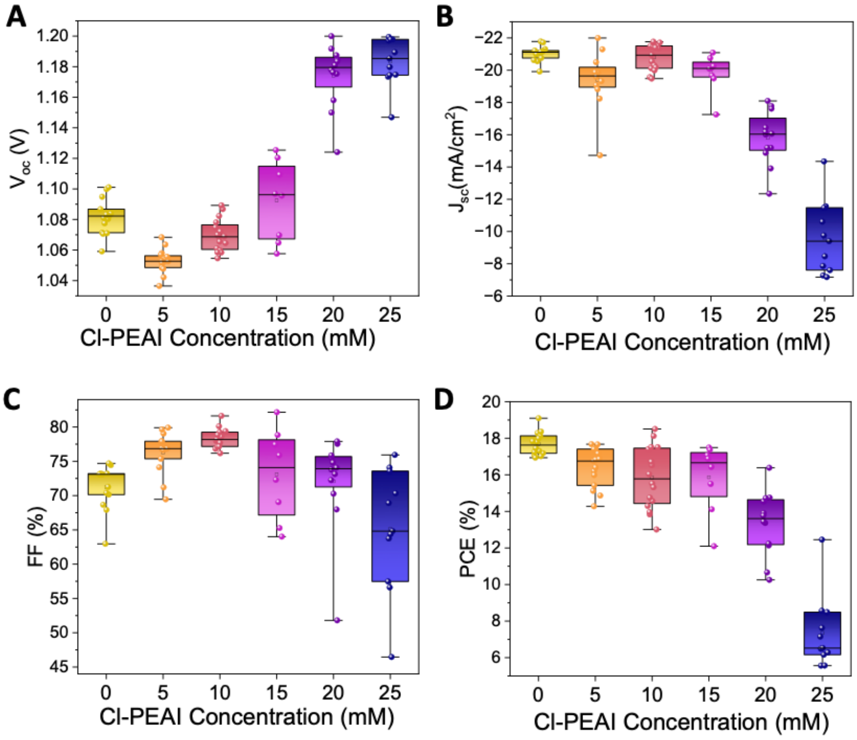

Figure S7: (A) V_{OC}, (B) J_{SC}, (C) FF, and (D) PCE of solar cells prepared via the interlayer method as a function of Cl-PEAI concentration ranging from 0 mM to 25 mM. The statistics, including the interquartile range, median, and minima and maxima excluding outliers, were calculated using 2 substrates per condition, with 8 solar cells per substrate.

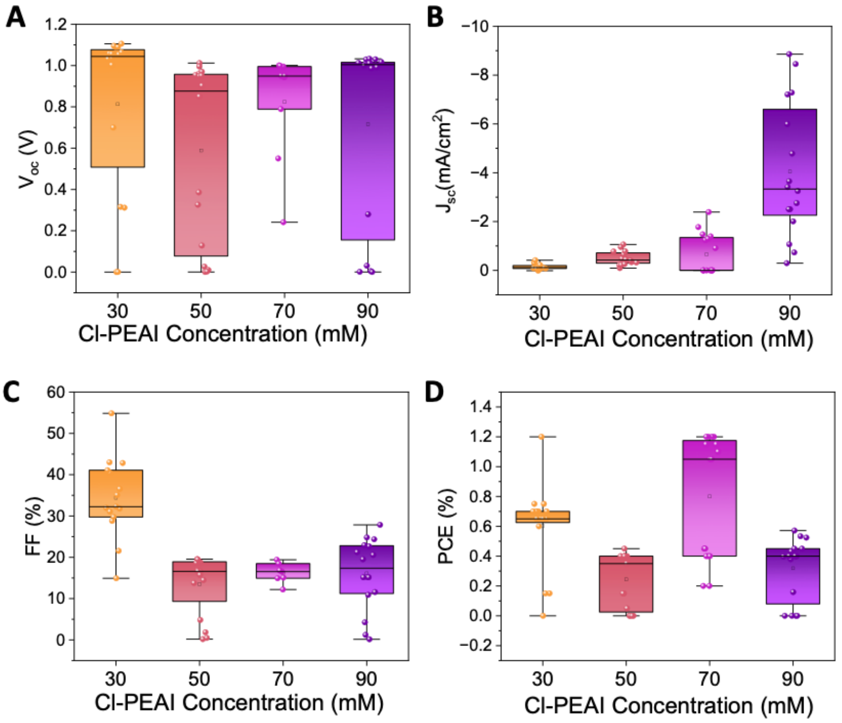

Figure S8: (A) V_{OC}, (B) J_{SC}, (C) FF, and (D) PCE of solar cells prepared via the interlayer method as a function of Cl-PEAI concentration ranging from 30 mM to 90 mM.

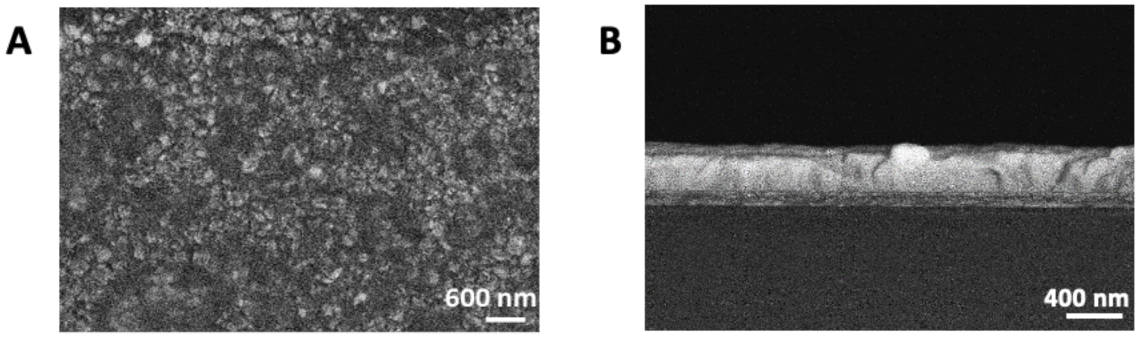

Figure S9: (A) Top-view and (B) cross-sectional SEM images of an interlayer sample with 20 mM concentration of Cl-PEAI.

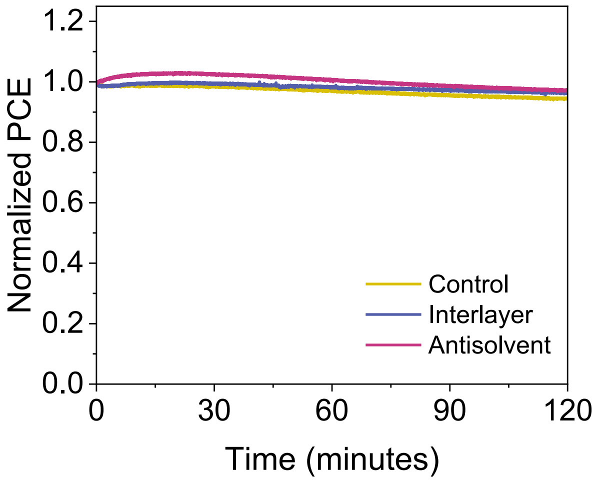

Figure S10: Maximum power point (MPP) tracking of the control and the films with 2 mM of Cl-PEAI deposited via the interlayer and antisolvent methods.
